# In vitro and in silico insights into antimicrobial and anticancer activities of novel imidazo[2,1-b][1,3,4]thiadiazoles

**DOI:** 10.1038/s41598-024-83498-x

**Published:** 2024-12-30

**Authors:** Deepika Dwarakanath, Yogeesha N. Nayak, Ananda Kulal, Samyak Pandey, K Sreedhara Ranganath Pai, Santosh L. Gaonkar

**Affiliations:** 1 Department of Chemistry, Manipal Institute of Technology, Manipal Academy of Higher Education, Manipal, Karnataka 576104 India; 2 Biological Sciences Division, Poornaprajna Institute of Scientific Research, Bengaluru Rural, 562110 Karnataka India; 3 Department of Pharmacology, Manipal College of Pharmaceutical Sciences, Manipal Academy of Higher Education, Manipal, Karnataka 576104 India

**Keywords:** Biochemistry, Biological techniques, Cancer, Chemistry

## Abstract

This study explores the design, synthesis, and evaluation of a novel series of isobenzofuran-based imidazo[2,1-b][1,3,4]thiadiazole derivatives, targeting their antimicrobial and anticancer properties. These compounds integrate the pharmacologically significant 1,3,4-thiadiazole and imidazole moieties, which are known for their potential in drug development, although imidazo[2,1-b][1,3,4]thiadiazole-based drugs are not yet available on the market. Therefore, the aim of this study is to develop novel derivatives that could serve as promising candidates for future therapeutic applications. The derivatives were synthesized in two steps and thoroughly characterized using IR, ^1^H NMR, ^13^C NMR, and mass spectrometry. All the derivatives had shown fairly good antimicrobial activity against four microorganisms (*Escherichia coli, Staphylococcus aureus*, *Mycobacterium smegmatis* and *Candida albicans*) with minimum inhibition concentration’s ranging from 0.14 to 0.59 mM. The anticancer activity of the compounds against MCF-7 cell lines showed promising activity, where three derivatives, **3a**, **3c** and **3d** exhibited better inhibition than the standard, cisplatin. The highest anticancer activity was shown by the derivative **3c** with an IC_50_ value of 35.81 μM. Molecular docking was studied to determine the docking poses and binding interaction of the derivatives with the protein bearing PDB: 5BNS and 3ZNR; ADME properties of the derivatives are also inferred which gives insights into the bioavailability. The molecular dynamics simulation of the derivative **3c** with HDAC7 protien (PDB: 3ZNR) was evalauted to determine the stability of the interaction between the protein and the ligand.

**Supplementary Information:**

The online version contains supplementary material available at 10.1038/s41598-024-83498-x.

## Introduction

**Fig. 1:**
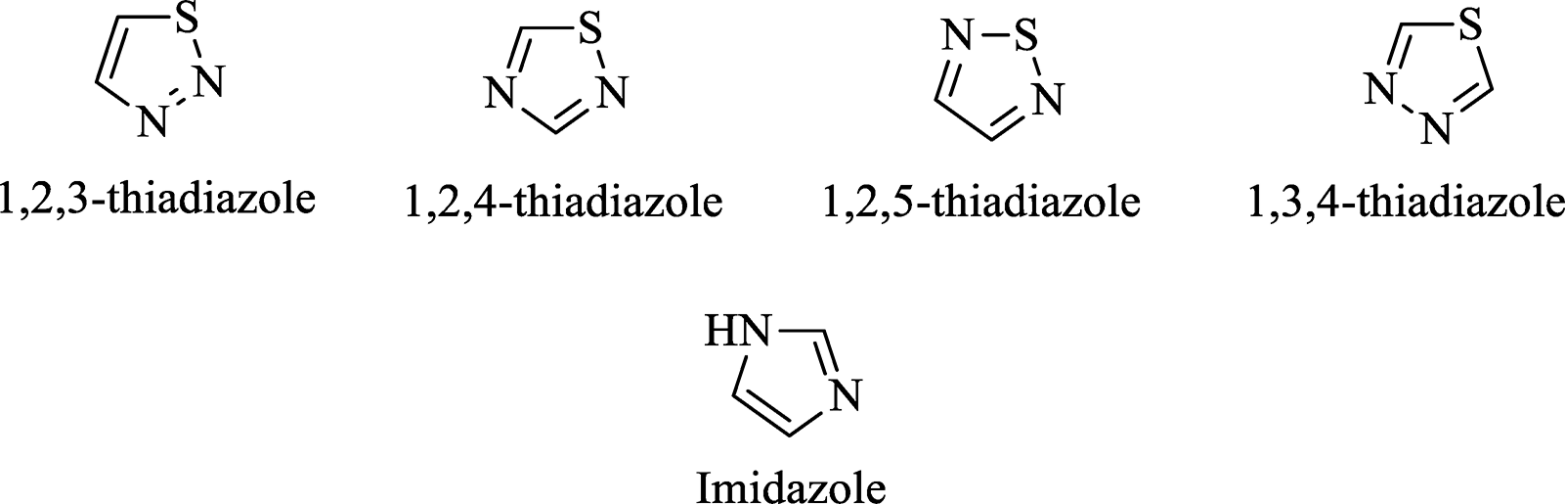
Structures of thiadiazoles and imidazole.

Heterocycles are a prominent sub-discipline within organic chemistry, characterized by the presence of at least one heteroatom, such as oxygen, nitrogen, or sulfur, within their ring structure. These compounds can form rings of various sizes, including three, four, five, and multi-membered configurations. Heterocycles containing nitrogen and sulfur are particularly significant in medicinal chemistry due to their biological activity and therapeutic potential^1–3^. Among them, five-membered heterocycles serve as essential building blocks in the development of numerous drugs currently used to treat a wide range of diseases. A notable class within this category is the 1,3,4-thiadiazoles, which have gained considerable attention for their diverse pharmacological properties with good antimicrobial^4,5^ anticancer^6^, antifungal^7^, anti-inflammatory^8,9^, antitubercular^10^, and antiviral^11^ activities. They are built of one sulphur and two nitrogen atoms. They exist in isomers such as 1,2,3-thiadiazole, 1,2,4-thiadiazole, 1,2,5-thiadiazole and 1,3,4-thidiazole (Fig. 1). The common isomer 1,3,4-thiadiazole shows a wide range of pharmaceutical properties because of either the = N-C-S- moiety or the aromatic nature of the ring. They are less toxic and exhibit good stability in vivo^5,12^.

**Fig. 2:**
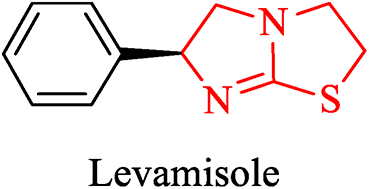
Drug bearing imidazo[2,1-b]thiazole core used to treat worm infections.

Likewise, imidazoles bearing two nitrogen atoms in their skeleton also possess various biological activities as they are comparatively more polar. The fusion of these two heterocycles yields another class of molecules, imidazo[2,1-b][1,3,4]thiadiazole scaffolds which have gained significance through the years^13–15^. The potential to lessen the detrimental effects of the cytotoxic drugs on the immune system also seems highly appealing, given that the imidazo[2,1-b]-1,3,4-thiadiazole system is somewhat comparable to Levamisole (Fig. 2), a well-known immunomodulator^16^. Derivatives bearing imidazo[2,1-b]-1,3,4-thiadiazole skeleton, display a wide range of medicinal properties such as anticancer^17^, antitubercular^18^, antibacterial^19^, antifungal^20^, anticonvulsant^21^, antioxidant^22^, tubulin inhibitor^23^, anti-inflammatory^24^, antihypertensive^25^, diuretic^26^, and antiviral^27^. Owing to these properties they are being exploited further in the medicinal field to get the best drug candidates.

Focusing on the drugs, antibiotic resistance is becoming a pressing worldwide health issue. Antibiotic-resistant illnesses have serious side effects, including higher death rates, longer disease duration, more medical expenses, and fewer treatment alternatives. The most serious problem is the rise of bacterial resistance to common antibiotics. The worrisome rise in the number of multi-drug resistant strains seen globally prompted the World Health Organization (WHO) to designate this phenomenon as a major health concern^28–31^. Cancer is another significant health concern. The term “cancer” is used to describe a wide range of illnesses that can begin in almost any organ or tissue in the body and spread to other organs as a result of abnormal cells proliferating out of control, attacking neighboring body parts, or migrating to other organs^32,33^. The metastatic cancer in the recent decade has increased with respect to different cancers, which kills about ten million people yearly^34^. Chemotherapy’s primary drawback is its incapacity to differentiate between cancerous and healthy cells, which lead to severe toxicity and adverse consequences. The therapy of cancer has drastically changed during the last 20 years, moving from broad-spectrum cytotoxic medications to targeted cancerous cells. In this case, the normal healthy cells are not affected but the cancerous cells are targeted^35,36^. Therefore, there is ongoing need for the development of novel compounds that can be evaluated for their potential to combat these debilitating diseases, offering hope for more effective treatments.

**Fig. 3:**
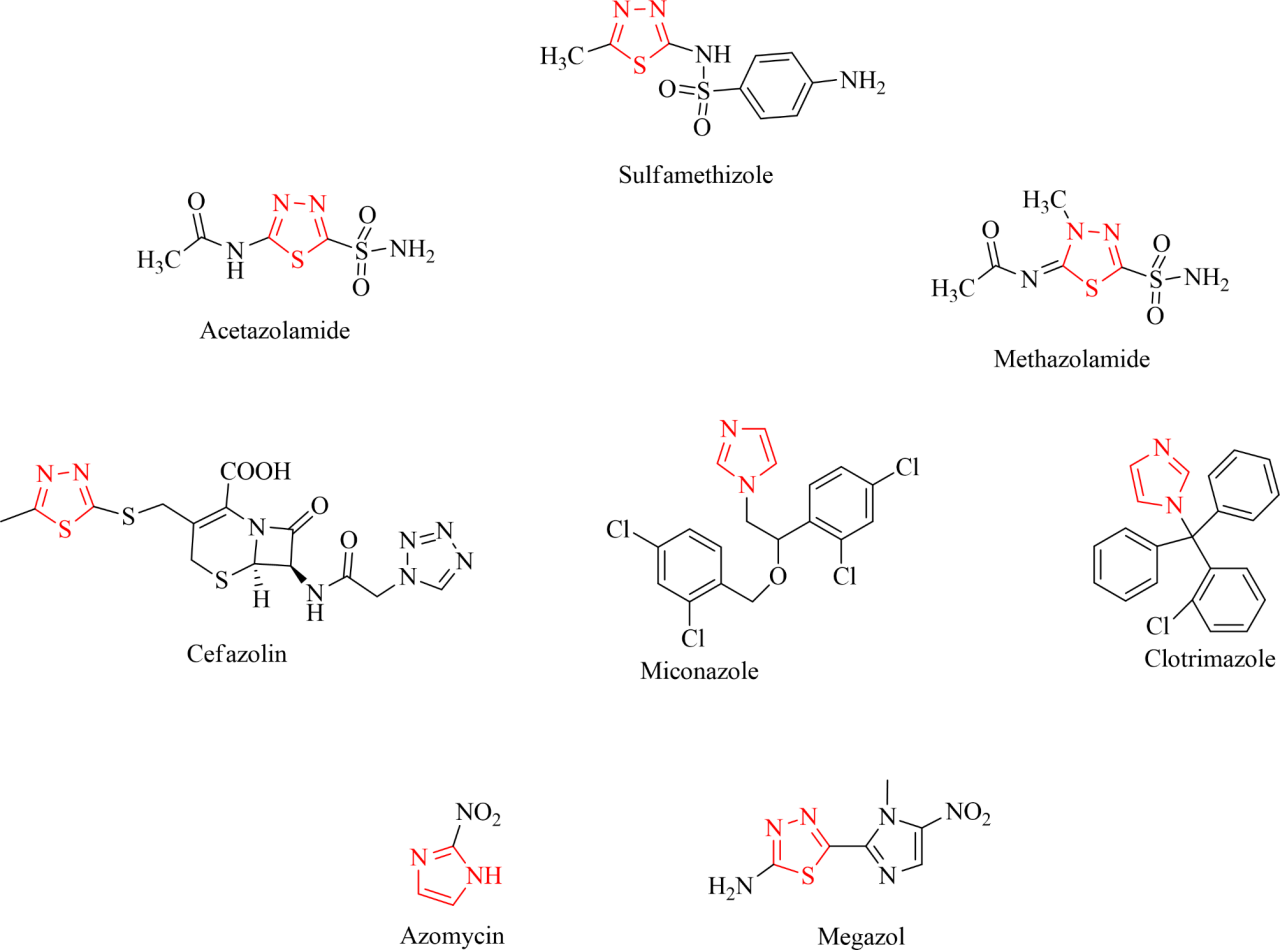
Drugs containing 1,3,4-thiadizole and imidazole scaffold.

Some of the drugs available in the market with 1,3,4-thiadiazole and imidazole moieties are represented in Fig. 3^37^. The core imidazo[2,1-b][1,3,4]thiadiazole has the potential to exhibit varies therapeutic activities but not many drugs with this scaffold are available in the market. To bridge this gap, in this work, isobenzofuran based imidazo[2,1-b][1,3,4]thiadiazole derivatives were designed, synthesized and their antimicrobial and anticancer properties were evaluated.

**Fig. 4:**
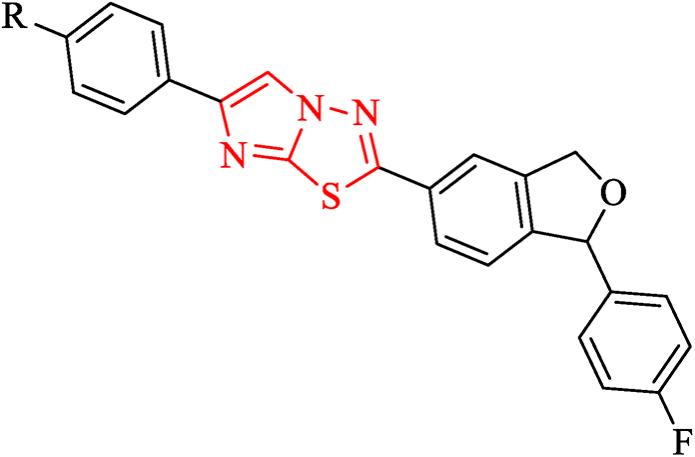
Design of imidazo[2,1-b][1,3,4]thiadiazole.

Design of drugs in the present study, includes fused imidazole and 1,3,4-thiadiazole ring, as represented below in Fig. 4.

## Results and discussion

### Chemistry

**Scheme 1:**
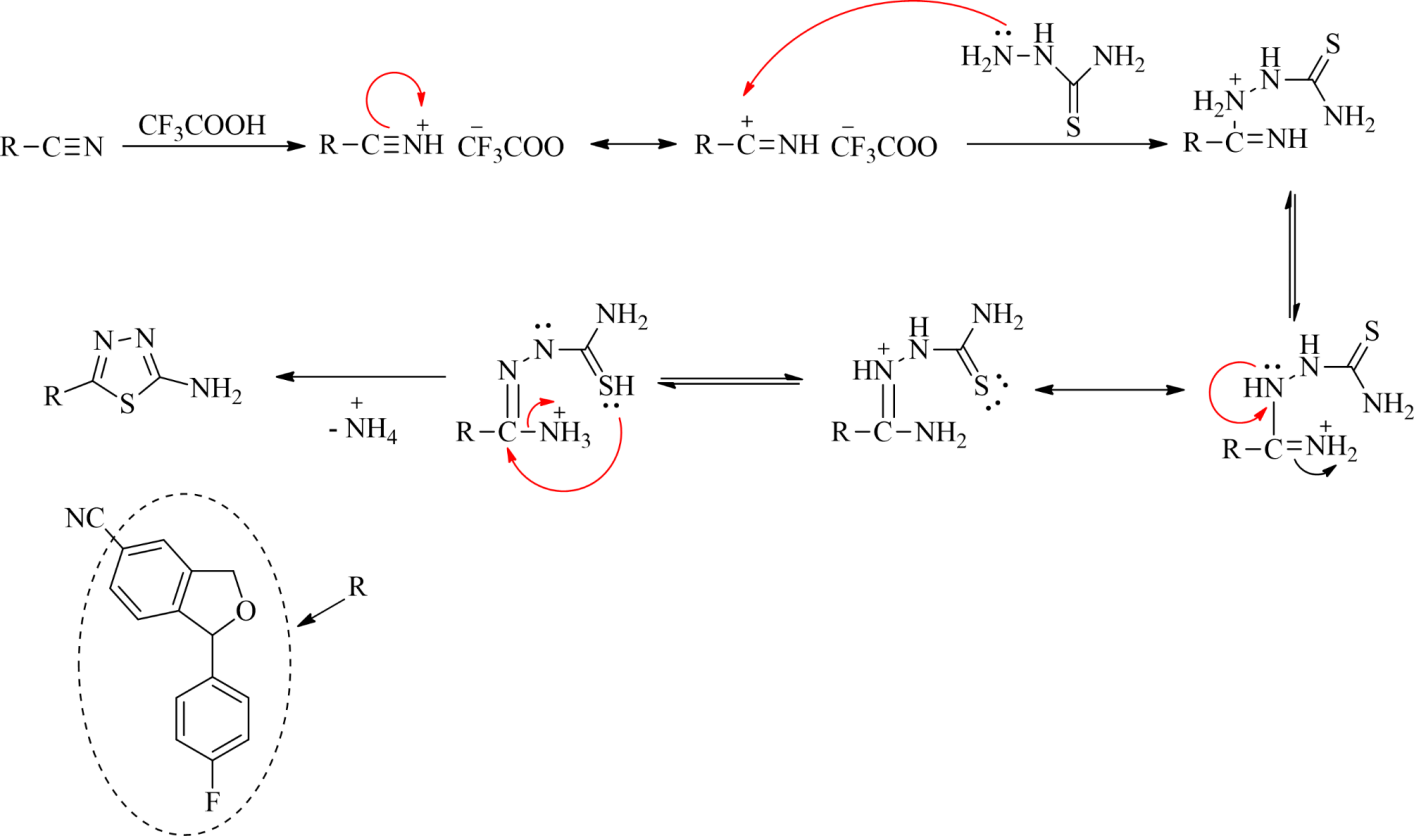
Plausible mechanism for the synthesis of 5-(1-(4-Fluorophenyl)-1,3-dihydroisobenzofuran-5-yl)-1,3,4-thiadiazol-2-amine (**2**).

**Scheme 2:**
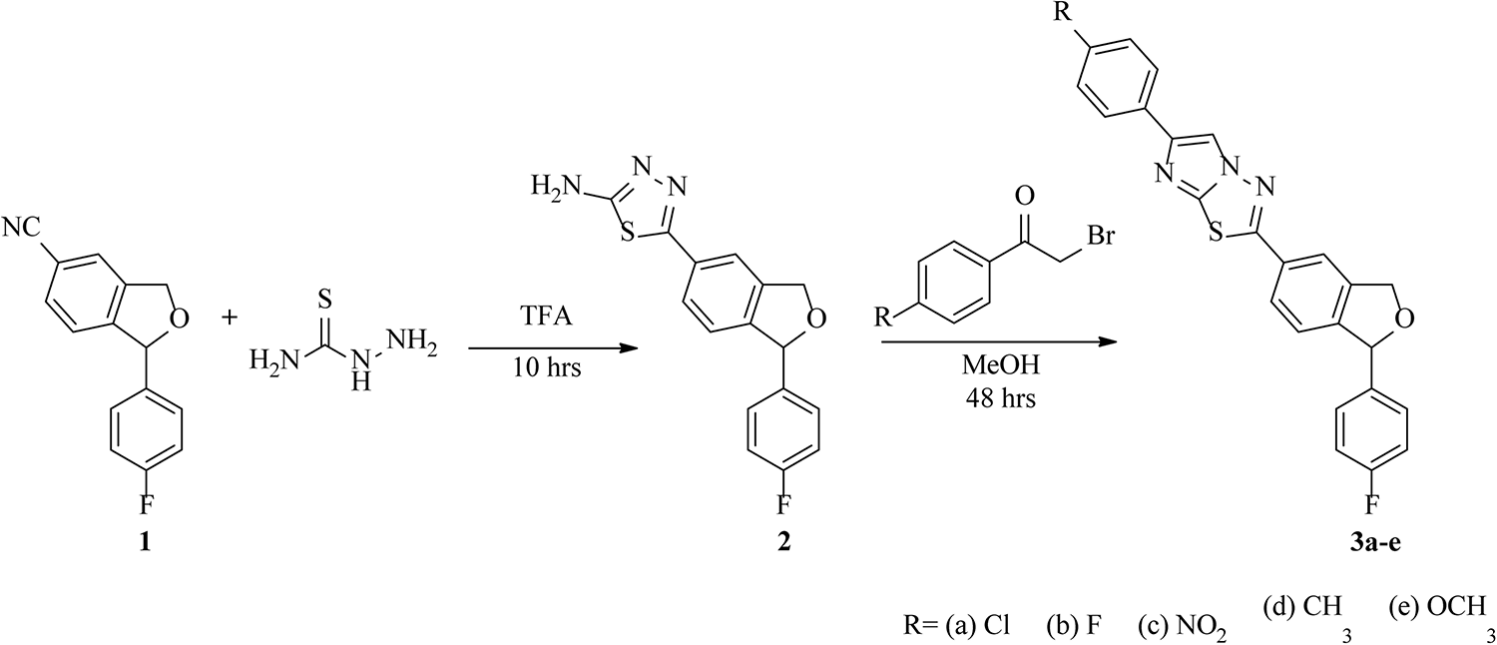
Synthesis of imidazo[2,1-b][1,3,4]thiadiazoles derivatives (**3a-3e**).

**Scheme 3:**
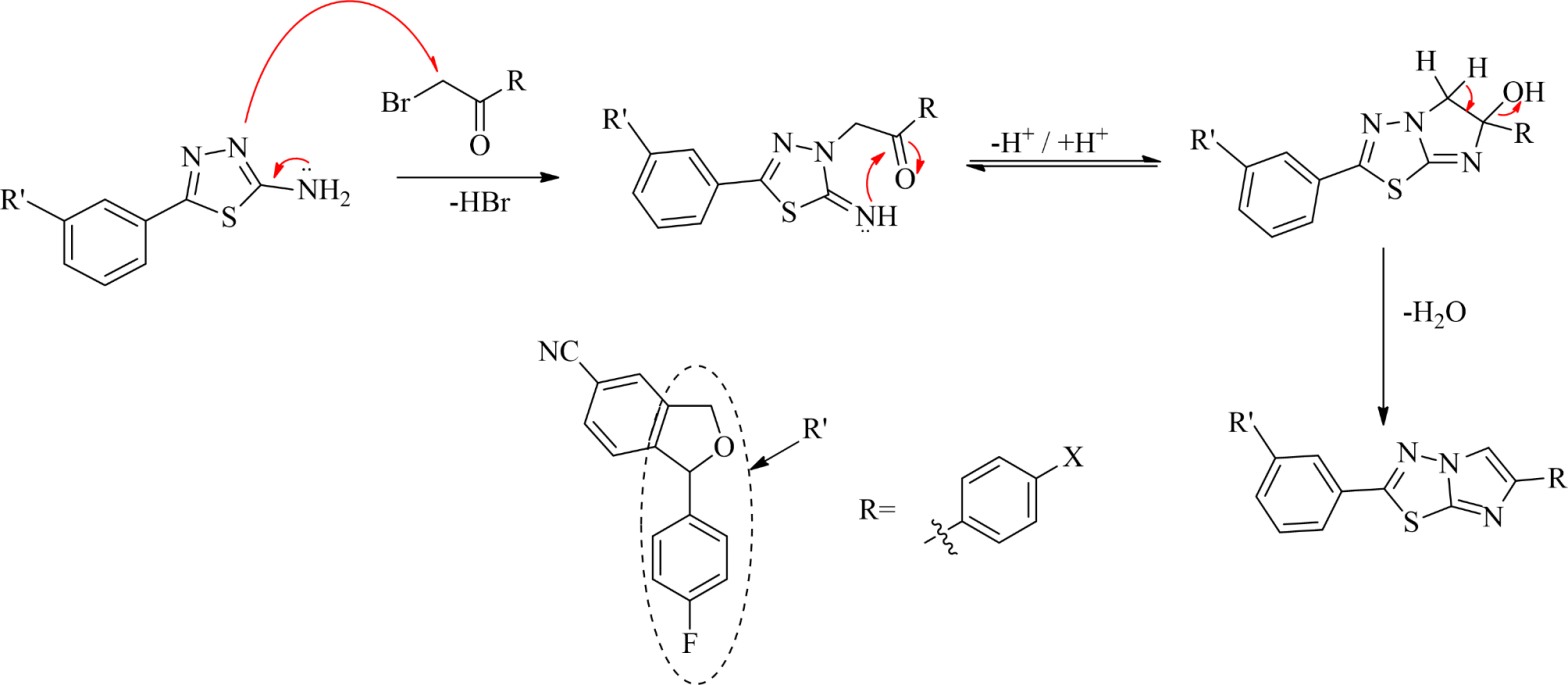
Plausible mechanism for the synthesis of imidazo[2,1-b][1,3,4]thiadiazole derivatives (**3a-3e**).

1-(4-Fluorophenyl)-1,3-dihydroisobenzofuran-5-carbonitrile (**1**) was synthesized according to the literature procedure^38^. Equimolar ratios of **1** and thiosemicarbazide were reacted in trifluoroacetic acid (TFA) for 10 h at 60 ˚C. After completion, the reaction mixture was cooled and neutralized with ammonium hydroxide to afford 5-(1-(4-Fluorophenyl)-1,3-dihydroisobenzofuran-5-yl)-1,3,4-thiadiazol-2-amine (**2**). Here, TFA acted as a solvent and catalyst for the reaction which aids the nucleophilic attack of thiosemicarbazide on iminium carbon. The plausible mechanism for the formation of **2** is represented in Scheme 1. This mixture was further reacted with different phenacyl bromides in methanol at reflux to yield in the target derivatives, imidazo[2,1-b][1,3,4]thiadiazoles (**3a-3e**) which were recrystallized in methanol. In both the steps, thin layer chromatography (TLC) was used to check the progress of the reaction and as primary indicator for the formation of products. The synthetic route is represented in Scheme 2. The plausible mechanism for the formation of target derivatives is shown in Scheme 3.

The infrared (IR) spectroscopy revealed peaks around 3120–3150 cm^-1^ and 2830–2880 cm^-1^ indicating aromatic C-H and aliphatic C-H streching, respectively. 1602–1604 cm^-1^ corresponds to C = N absorption band and aromactic C = C streching can be seen around 1467–1487 cm^-1^. Melting points of all compounds were found to be above 200 ˚C. The proton NMR gives further details about the formation of the derivatives by confirming the presence of aromatic hydrogens in the region of 7.00–9.04 ppm. Two doublet signals at 5.21–5.40 ppm, for methylene protons of isobenzofuran ring is observed which is due to coupling of diastereotopic protons. In all the derivatives, a single peak at 6.26 ppm corresponds to the CH proton of isobenzofuran. Methyl protons of compounds **3d** and **3e** can be observed at 2.32 ppm and 3.79 ppm, respectively. The number of carbon peaks in the ^13^C- NMR corresponds to the total number of carbons present in the derivatives. The isobenzofuran methylene carbon in all derivatives were observed around the range 71.73–71.80 ppm; likewise the CH carbon of isobenzofuran ring is in the range of 84.36–84.52 ppm. The methyl and methoxy carbon of derivatives **3d** and **3e** are observed at 21.31 and 54.31 ppm respectively. The carbon attached to fluoro group is seen around 161.16–163.52 ppm. The fused imidazole-thiadiazole carbon is seen around 135.78 ppm. The other carbon peaks corresponds to the aromatic carbons in the derivatives. Further, mass spectrometry was done to validate mass of the synthesized derivatives which was found to be in good agreeance with theoretical value.

### Molecular docking

The protein chosen for antibacterial activity is associated with initiation of fatty acid biosynthesis. Inhibition of this enzyme leads to reduced proliferation, increased cell death and induction of neural differentiation. The histone deacetylase 7 (HDAC7) protein is mainly involved in epigenetic repression, transcriptional regulation and cell cycle progression; this a worthy target to arrest growth of cancer cells. Therefore, these proteins were chosen for the current study.

### Docking of derivatives with Fabh protein (PDB: 5BNS)

**Fig. 5:**
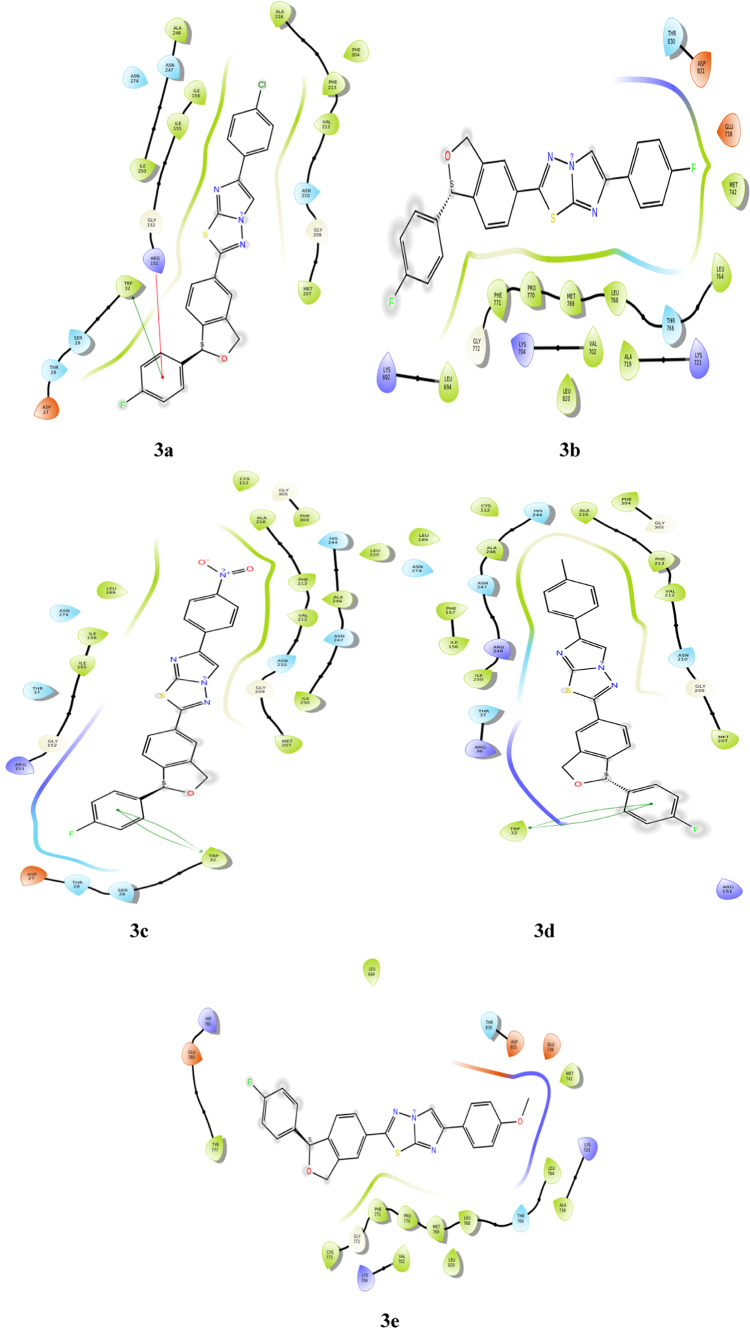
Docking of compounds **3a-3e** into the Fabh protein.

The docking pose of the ligands with Fabh protein was established as shown in Fig. 5. The phenyl ring attached to isobenzofuran of derivtaives **3a**, **3c** and **3d** form pi-pi stacking interaction with amino acid **TRP 32**. The same phenyl ring in derivative **3a**, interacts with **ARG 151** through pi-cation bond. Non bonding interactions are observed in derivative **3b** such as polar and non polar; solvent exposure can be observed around 4-fluoro phenyl group tethered to isobenzofuran. Derivative **3e** exhibits no bonding interactions but charged and non polar non boning interactions can be observed.

### Docking of derivatives with human HDAC7 (PDB ID: 3ZNR)

**Fig. 6:**
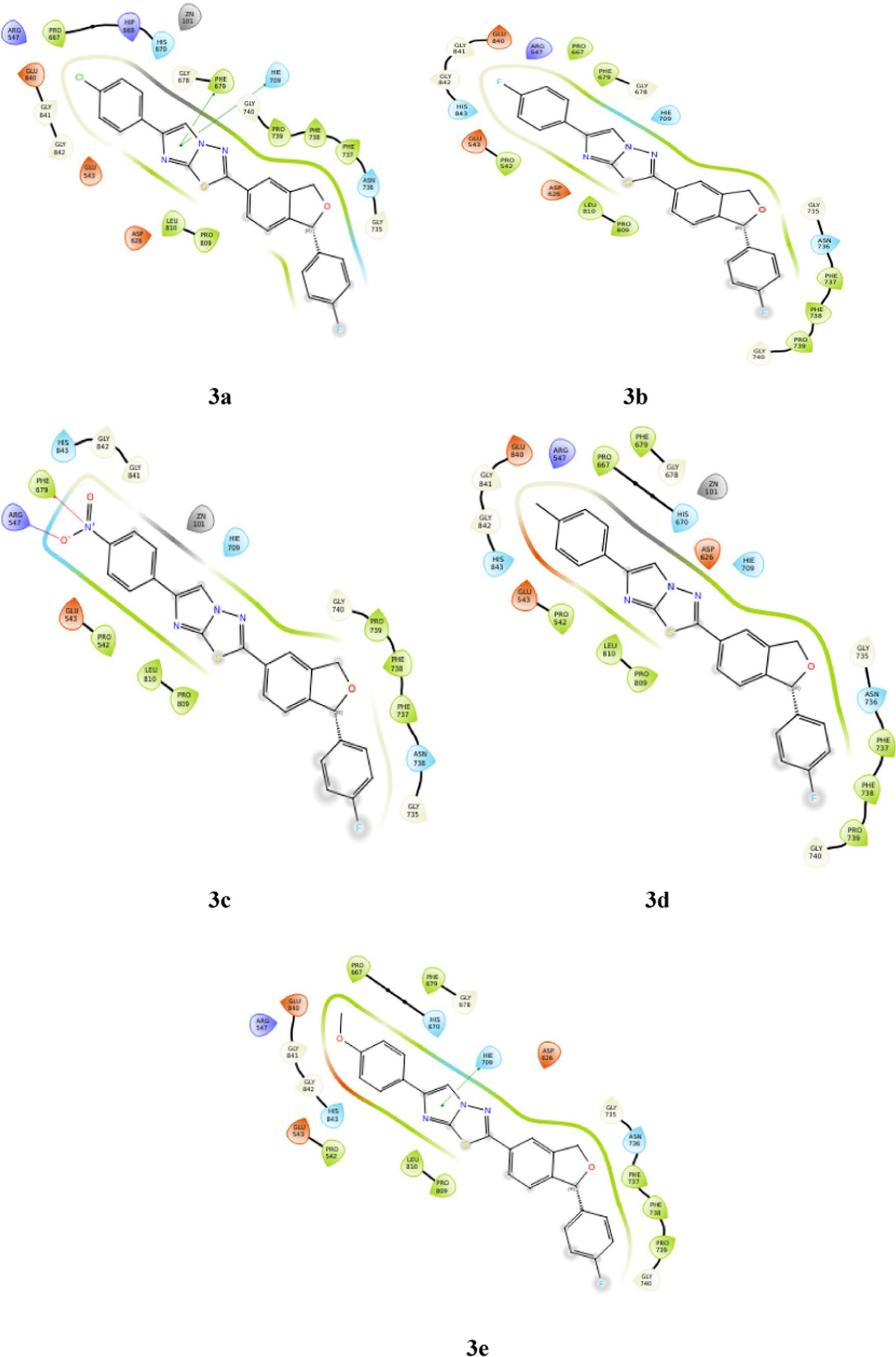
Docking of compounds **3a-3e** into the HDAC7 protein.

The fused imidazole ring forms two pi-pi stacking interactions with **PHE 679** and **HIE 709** in derivative **3a** along with non polar interactions. Derivatives **3b** and **3d**, shows no bonding interactions but non bonding interactions such as non polar and charged interactions can be observed. The nitrogen of the nitro group in derivative **3c**, forms pi-cation interaction with amino acid **PHE 679**; and the oxygen of nitro group forms salt bridge with **ARG 547**. Apart from this non polar interaction can be seen in the same derivative. In derivative **3e**, pi-pi stacking interaction can be seen between **HIE 709** and imidazole ring. The docking poses are represented in Fig. 6.

### ADME studies

The molecular weights of the synthesized compounds are well within the range (< 500). There are no hydrogen bond donors in all derivatives and about 4 hydrogen bond acceptors in all the five derivatives. The water/gas partition coefficient reveals the hydrophilicity or hydrophobicity of the compounds and are within the expected range. All the synthesized compounds follow Lipinski’s rule of five without violations (Reference Values (RV) are also given) (Table 1).

**Table 1:** Lipinksi’s rule of five pararmeters **3a-3e.**

Compound	Mol. Wt	QPLogPo/W	H-donor	H-acceptor
3a	447	6.419	0	4.2
3b	431	6.153	0	4.2
3c	458	5.178	0	4.2
3d	427	6.247	0	4.2
3e	443	5.980	0	4.95
RV	130–500	-2–6.5	0–6	2–20

Table 2 presents other parametrs that determine drug bioavailibility like solubility (QPLogS), percentage oral absorption, blood–brain barrier permeability (QPLogBB) and polar surface area (PSA). The solubility of the compounds are measured at 25 ˚C as a log of its molar concentration, here, all compounds are outside the recommended range indicating slightly poor solubility. However, the percentage oral absoption of all the derivatives are very good (100%) except derivative **3c** with 92%. The blood brain barrier permeability gives insight into the diffusion (distribution parameter) of the drug depending upon its binding affinity to blood protein; all derivatives are within the suggested range to be a BBB permeant. Compared to all the derivatives, **3c** has the lowest QPLogBB value. The total solvent accessible surface area (SASA) of the compounds is agreeable with range recommended. The Vander Waals surface area of oxygen and nitrogen atoms is represented as polar surface area which ranges from 37.465–82.464 and is within 200. The ADME data reveals that the synthesized compounds are in good agreement with the values advocated with respect to each parameter.

**Table 2:** ADME properties of derivatives **3a-3e**.

Compound	QPLogS	QPLogBB	%Abs	QPPCaco	SASA	PSA
3a	-8.299	0.560	100	4394.634	716.812	37.465
3b	-7.906	0.505	100	4393.196	701.763	37.468
3c	-7.624	-0.734	92.93	521.204	733.873	82.464
3d	-8.156	0.382	100	4397.221	725.687	37.468
3e	-7.691	0.323	100	4395.812	730.463	45.660
RV	-6.5–0.5	-3–1.2	Max. 100	< 25 poor > 500 good	300–1000	7–200

### Antimicrobial activity

The evaluation of the antimicrobial effects of the synthesized derivatives **3a-3e** against four microorganisms revealed that they displayed similar inhibitory effects. The minimum inhibitory concentrations (MICs) of derivatives **3b-3e** against *M. smegmatis* and *C. albicans* were found to be at the range of 0.14 mM and 0.27–0.30 mM, respectively. Derivatives, **3a** and **3c** have exhibited similar inhibition against *E.coli* and *S. aureus* with an MIC of 0.27–0.28 mM. Likewise, derivatives, **3b** and **3e** displayed MIC of 0.28–0.29 mM and 0.56–0.58 mM against *E. coli* and *S. aureus*, respectively. However, derivative **3a** showed a slightly different activity compared to other derivatives with respect to *M. smegmatis* and *C. albicans* with an MIC of 0.28 mM and 0.14 mM respectively. It can be inferred here that all the derivatives irrespective of the substituents show similar activity especially towards *M. smegmatis* and *C. albicans* probably due to similar structure of the derivatives. The standards used were ciprofloxacin for bacteria and fluconozole for fungus where both had MIC of 0.01 mM. Table 3 represents the MIC values for all the derivatives and standards in millimolar concentration.

**Table 3:** Minimum inhibitory concentration of compounds **3a-3e** against microbial strains.

Compounds	Minimum Inhibitory Concentration (mM)			
	*E. coli*	*S. aureus*	*M. smegmatis*	*C. albicans*
3a	0.28	0.28	0.28	0.14
3b	0.29	0.58	0.14	0.29
3c	0.27	0.27	0.14	0.27
3d	0.59	0.59	0.14	0.30
3e	0.28	0.56	0.14	0.28
Ciprofloxacin	0.01	0.01	0.01	–
Fluconazole	–	–	–	0.01

## Anticancer activity

**Fig. 7:**
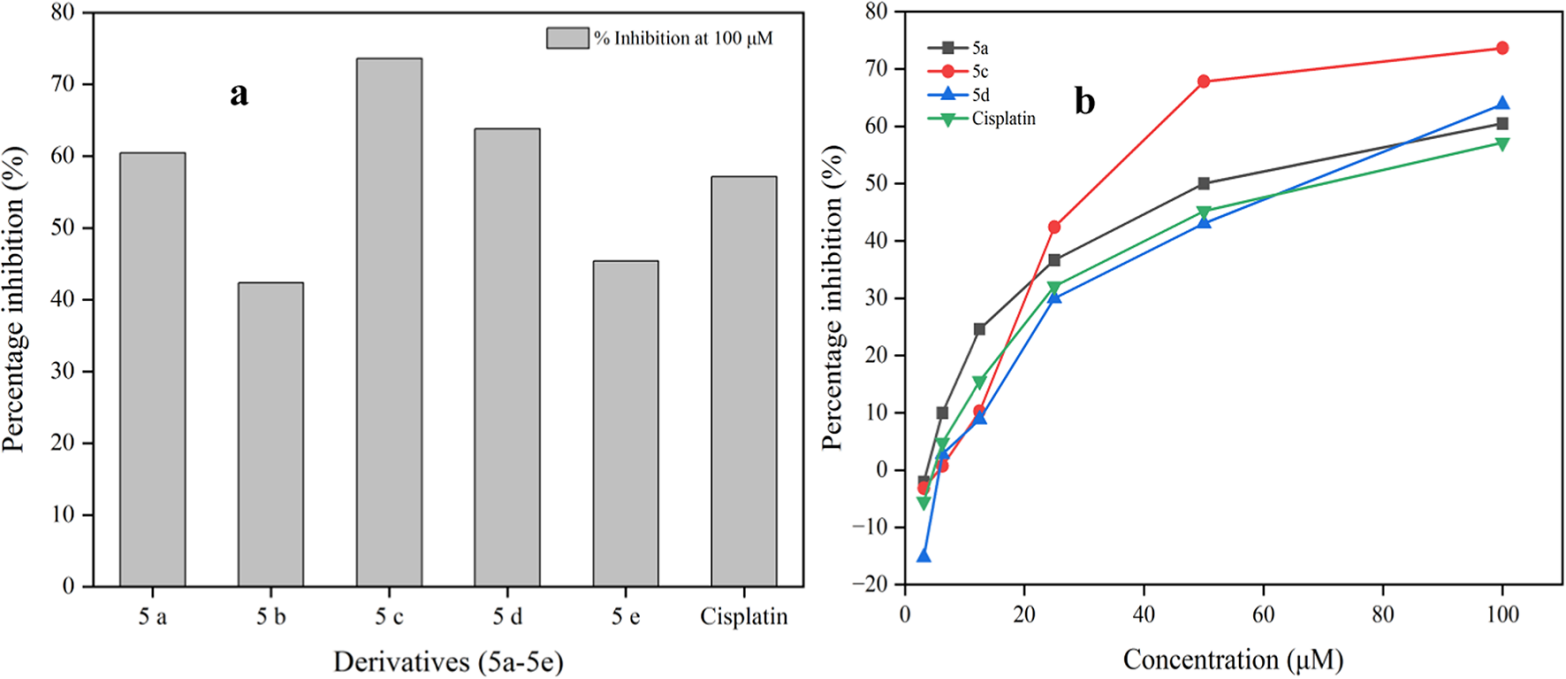
(**a**) Percentage inhibition of derivatives **3a-3e** and cisplatin (**b**) Percentage inhibition of derivatives **3a**, **3c**, **3d** and cisplatin at various tested concentrations (3.125–100 μM).

SRB assay was performed to evaluate the anticancer activity of the derivatives **3a-3e**. All six compounds including cisplatin as the standard were tested. Derivative **3c** exhibited excellent inhibition against MCF-7 cell line with an IC_50_ of 35.81 μM and percentage inhibition of 73% at 100 μM concentration. As seen in Fig. 7a derivatives, **3b** and **3e** display around 42–45% inhibition whereas derivatives, **3a** and **3d** show inhibition around 60–63%. The IC_50_ of derivatives, **3a** and **3d** are 52.62 μM and 61.74 μM, respectively. Although all derivatives show fair percentage inhibition, the IC_50_ of derivatives **3b** and **3e** were above 100 μM, i.e., beyond the experimental range. It is interesting to note that, derivatives **3a**, **3c** and **3d** show IC_50_ less than that of standard, cisplatin whose IC_50_ is 67.67 μM. Figure 7b represents percentage inhibition of derivatives **3a**, **3c**, **3d** and cisplatin at various tested concentrations—3.125 μM, 6.25 μM, 12.5 μM, 25 μM, 50 μM and 100 μM.

### Molecular dynamics (MD) simulation

**Fig. 8:**
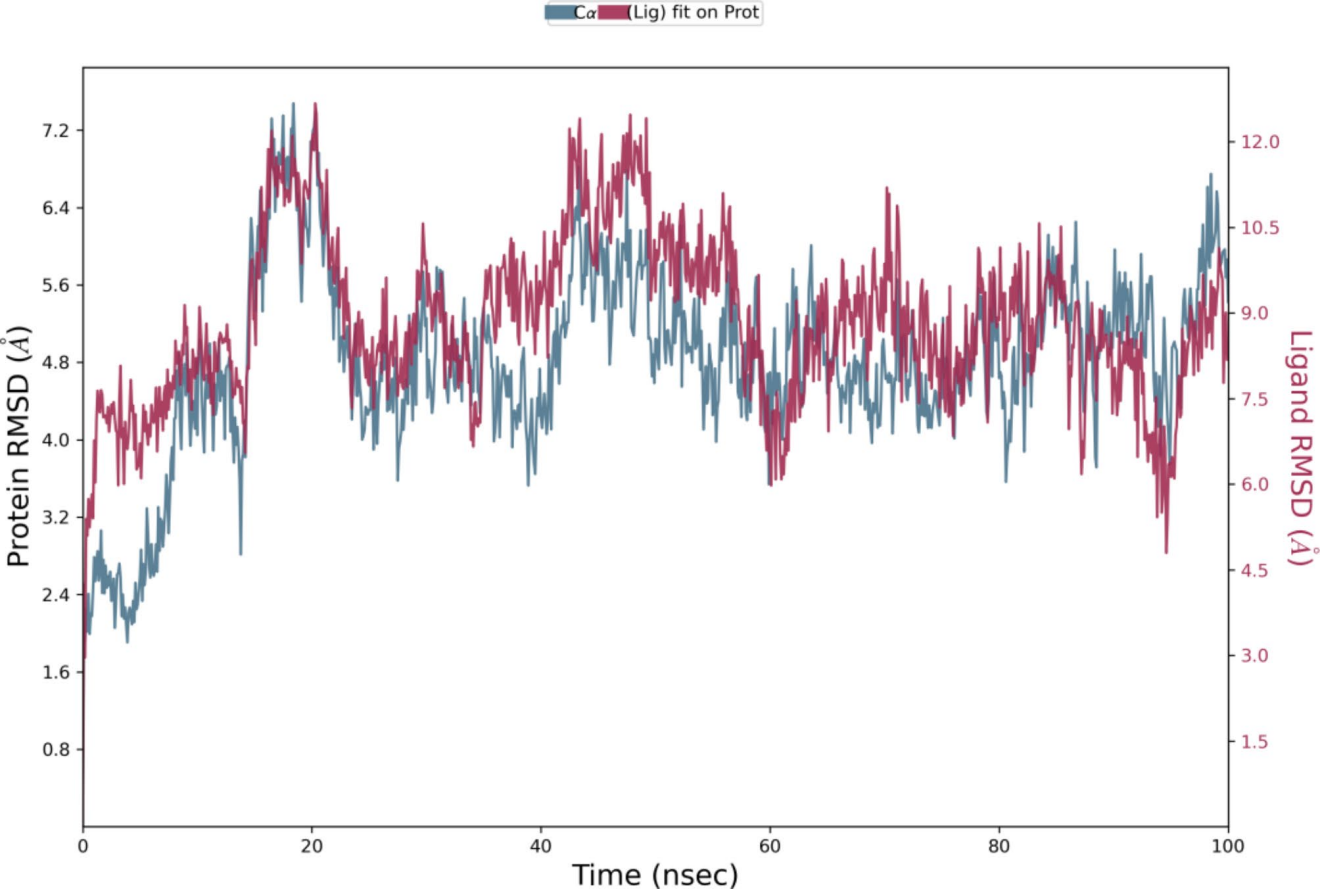
RMSD of the protein–ligand complex.

**Fig. 9:**
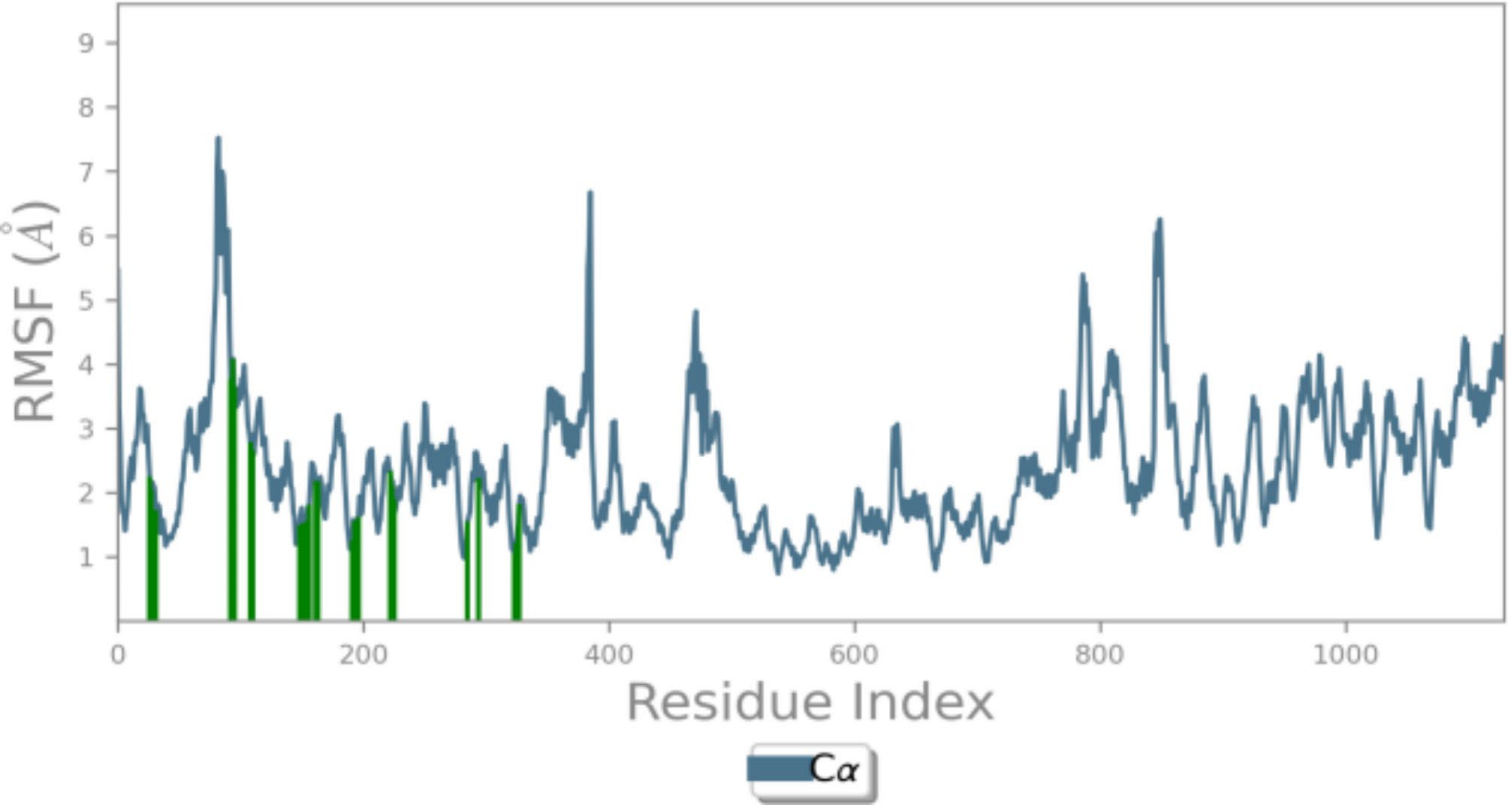
RMSF map of the protein–ligand complex (HDAC7-3a) during the 100 ns simulation.

**Fig. 10:**
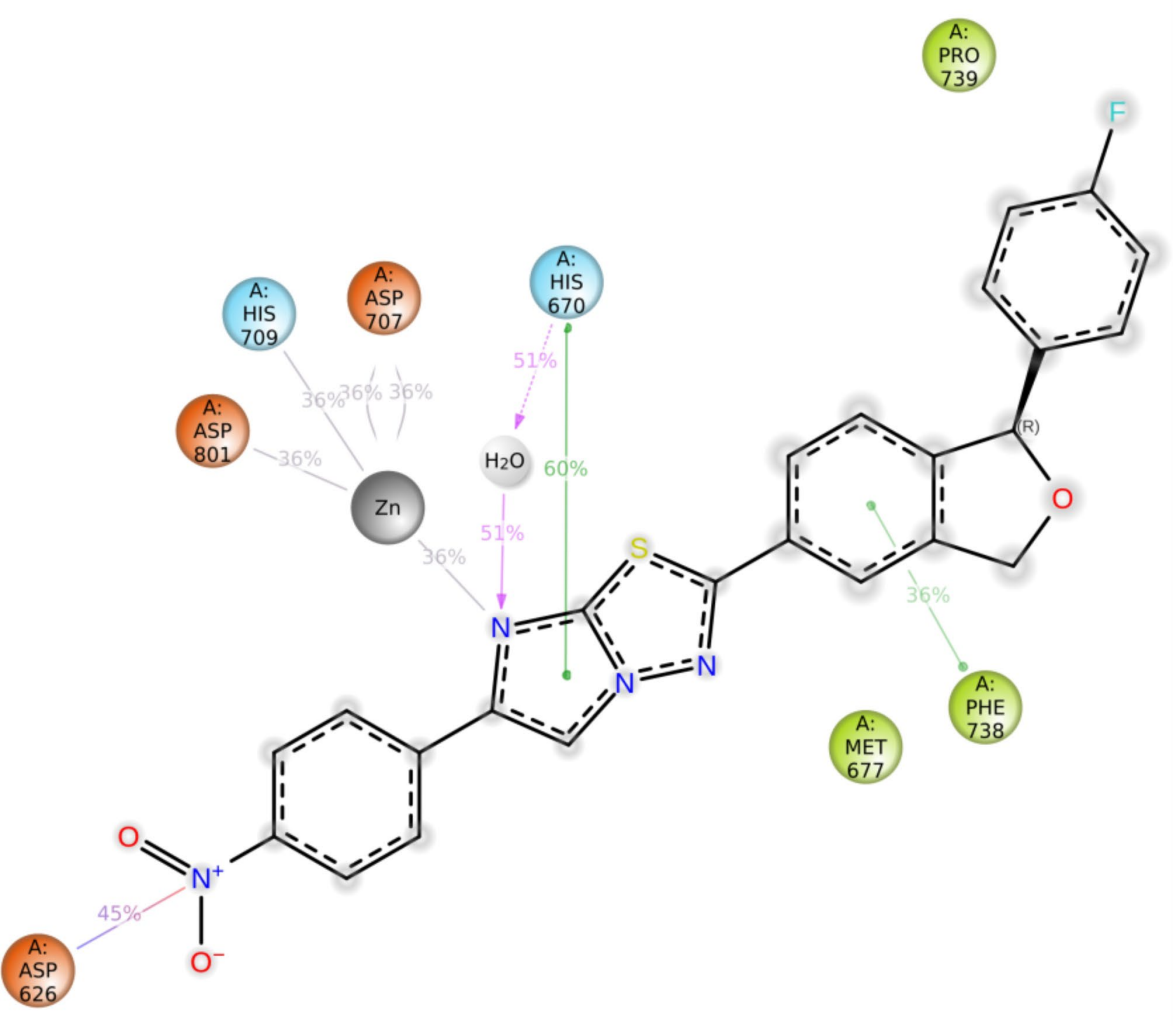
Ligand interactions with protein residues throughout the trajectory.

**Fig. 11:**
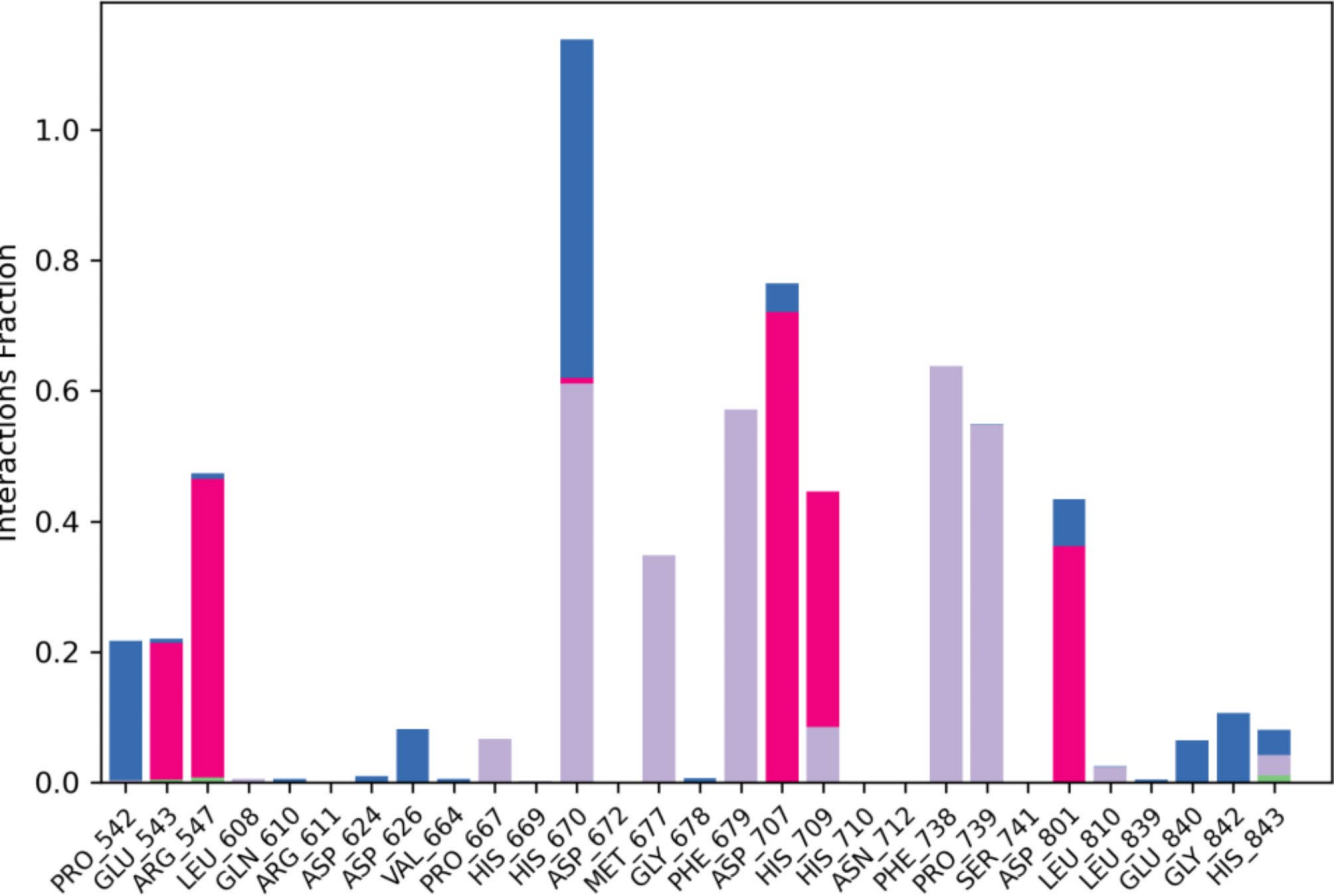
Histogram of protein‒ligand contacts during the 100 ns simulation.

MD simulation was carried out for derivative **3c** as it exhibited outstanding inhibition with an IC_50_ of 35.81 μM. This study was performed to evaluate the stability of the compound within the active site of the protein HDAC7 (PDB: 3ZNR) for 100 ns. As shown in Fig. 8, the Root Mean Sqaure Deviation (RMSD) for protein–ligand complex (HDAC7-3a) in the initial stage of simulation shows fluctuation upto 20 ns. However, the complex stabilises after 20 ns and the deviation was found to be around 2–2.5 Å. The RMSD of the ligand is lesser than the RMSD of the protein indicating that the ligand has not deviated much from the initial binding site. The Root Mean Square Fluctuation (RMSF) gives insight into the local changes that take place along the protein chain during MD. This is represented in Fig. 9 which displays the interaction between the protein residues and ligand. Figure 10 shows the MD ligand docking interaction along with percentage of interaction, here, the nitrogen of the nitro group forms salt bridge with **ASP 626** which was initially pi-cation interaction with **PHE 679**. Additionally, there are two pi-pi stacking interaction, one between imidazole ring and **HIS 670**; second between benzene ring of isobenzofuran and **PHE 738**. Interestingly, the nitrogen of the imidazole ring complexes with Zn metal present in the protein, although the percentage of interaction is less than 40%. The protien-ligand contacts diagram (Fig. 11) shows that there are a number of hydrophobic and ionic interactions, along with the highest hydrogen bonded interations mediated with water with amino acid residue **HIS 670**. This simulation thus explains the stability of the complex.

### Structure activity relationship (SAR) studies

In this series, the electron withdrawing and electron donating substitutents did not play a substantial role in determining the activities of the derivatives. The overall architecture of the compounds showed similar inhibition with respect to antimicrobial activity. Notably, derivtaives of chloro, **3a** and nitro, **3c** showed similar inhibition against all microbes. And all the derivtaives, except **3a** displayed same inhibition against *M. smegmatis* and *C. albicans*. With respect to anticancer activity, the nitro derivative at fourth position showed excellent inhibition, followed by the chloro derivative, **3a** with an IC_50_ of 52.62 μM and methyl derivative, **3d** with an IC_50_ of 61.74 μM. According to this trend, it can be said that the electron withdrawing groups display slightly better inhibition than electron donating groups; however the difference is not very significant.

## Materials and methods

### Chemistry

The solvents and chemical reactants required for the synthesis were procured from Sigma Aldrich. Completion of reaction in each step was tracked on thin layer chromatography (TLC) sheets. TLC plates were observed under UV light. Melting points of compounds were recorded using Thiel’s tube. The IR spectrum was obtained by Shimadzu FTIR. ^1^H NMR (in DMSO-d6) and ^13^C NMR (in CDCl_3_) spectra of all the compounds were recorded using Bruker AM 400 MHz NMR spectrometer. Waters Alliance E2695/HPLC-TQD Mass Spectrometer (LC–MS/MS) was used to determine the mass of the compounds.

### Experimental

#### Procedure for the synthesis of 5-(1-(4-Fluorophenyl)-1,3-dihydroisobenzofuran-5-yl)-1,3,4-thiadiazol-2-amine (2)

Equimolar ratio of isobenzofuran carbonitrile (**1**)^38^, and thiosemicarbazide were heated in 6 ml of TFA at 60 ˚C for 20 hours. The progress of the reaction was tracked on TLC. After completion, the reaction temperature was brought down to room temperature and poured into crushed ice. 1N Ammonium hydroxide was used to neutralize the acid; the resulting solid was filtered and washed with water several times and recrystallized using ethanol to result in 5-(1-(4-Fluorophenyl)-1,3-dihydroisobenzofuran-5-yl)-1,3,4-thiadiazol-2-amine (**2**) (80% yield)

#### Procedure for the synthesis of 2-(1-(4-Fluorophenyl)-1,3-dihydroisobenzofuran-5-yl)-imidazo[2,1-b][1,3,4]thiadiazoles derivatives (3a-3e)

Typical procedure: α-bromoketones and 2-amino-1,3,4-thiadiazole derivative **(2)** were taken in equal ratio and refluxed in methanol for 48 hours. The obtained precipitate was filtered at hot and washed with hot methanolic solution to yield in imidazo[2,1-b][1,3,4]thiadiazoles derivative **(3a)**. Other derivatives (**3b-3e**) were synthesized using the same procedure.

**Spectral data:**

*6-(4-Chlorophenyl)-2-(1-(4-fluorophenyl)-1,3-dihydroisobenzofuran-5-yl)imidazo[2,1-b][1,3,4]thiadiazole* (**3a**): Off white solid. 60% yield. IR: ν_max_ (cm^-1^): 3127 (Ar C-H), 2864 (Aliphatic C-H), 1602 (C = N), 1436 (Ar C = C); ^1^H-NMR (DMSO-*d*_*6*_*,* 400 MHz) δ, ppm (*J*, Hz): 8.81 (1H, s, ArH); 7.99 (1H, s, ArH); 7.91 (1H,s, ArH); 7.85 (2H, d, *J* = 16, ArH); 7.49 (2H, d,*J* = 16, ArH); 7.43 (2H, d, *J* = 8, ArH); 7.21–7.34 (3H, m, ArH); 6.26 (1H, s, CH); 5.36 (1H, d, *J* = 12, CH_2_); 5.21 (1H, d, *J* = 8, CH_2_).^13^C-NMR (100 MHz, CDCl_3_) δ (ppm): 162.50, 160.58, 145.68, 143.89, 141.77, 140.01, 135.78, 133.64, 128.62, 127.87, 126.97, 126.61, 126.04, 122.53, 118.94, 114.83, 108.86, 84.37, 71.80. ES^+^ (m/z) = 448.4 (M + H)^+^.

*6-(4-Fluorophenyl)-2-(1-(4-fluorophenyl)-1,3-dihydroisobenzofuran-5-yl)imidazo[2,1-b][1,3,4]thiadiazole* (**3b**): Off white solid. 60% yield. IR: ν_max_ (cm^-1^): 3218 (Ar C-H), 2864 (Aliphatic C-H), 1604 (C = N), 1487 (Ar C = C); ^1^HNMR (DMSO-*d*_*6*_*,* 400 MHz) δ, ppm: 8.74 (1H, s, ArH); 7.98 (1H, s, ArH); 7.94–7.91 (2H, m, ArH); 7.86 (1H, d, J = 12, ArH); 7.45 (2H, t, J = 16, ArH); 7.19–7.28 (5H, m, ArH); 6.26 (1H, s, CH); 5.39 (1H, d, J = 12, CH_2_); 5.22 (1H, d, J = 16, CH_2_). ^13^C-NMR (100 MHz, CDCl_3_) δ (ppm): 163.00, 162.66, 160.55, 160.21, 160.01, 144.85, 144.74, 144.10, 139.72, 135.96, 135.93, 129.09, 128.94, 128.91, 127.87, 127.78, 125.80, 125.72, 125.63, 122.23, 118.47, 114.79, 114.78, 114.58, 114.57, 108.04, 84.37, 71.74. ES^+^ (m/z) = 432.4 (M + H)^+^

*2-(1-(4-Fluorophenyl)-1,3-dihydroisobenzofuran-5-yl)-6-(4-nitrophenyl)imidazo[2,1-b][1,3,4]thiadiazole* (**3c**): pale yellow solid. 65% yield. IR: ν_max_ (cm^-1^): 3132 (Ar C-H), 2877 (Aliphatic C-H), 1600 (C = N), 1515 and 1340 (NO_2_), 1467 (Ar C = C); ^1^HNMR (DMSO-*d*_*6*_*,* 400 MHz) δ, ppm: 9.04 (1H, s, ArH); 8.30 (2H, d, J = 8, ArH); 8.15 (2H, d, J = 8, ArH); 8.00 (1H, s, ArH); 7.87 (1H, d, J = 8, ArH); 7.41–7.45 (2H, m, ArH); 7.19–7.27 (3H, m, ArH); 6.26 (1H, s, CH); 5.40 (1H, d, J = 16, CH_2_); 5.22 (1H, d, J = 12, CH_2_). ^13^C-NMR (100 MHz, CDCl_3_) δ (ppm): 161.16, 145.89, 145.17, 145.08, 143.30, 139.84, 139.00, 135.85, 128.79, 127.86, 127.78, 125.79, 124.38, 123.29, 122.34, 118.63, 114.81, 114.59, 110.35, 84.37, 71.73. (ES^+^ (m/z) = 459.2 (M + H)^+^

*2-(1-(4-Fluorophenyl)-1,3-dihydroisobenzofuran-5-yl)-6-(p-tolyl)imidazo[2,1-b][1,3,4]thiadiazole* (**3d**): Off white solid. 75% yield. IR: ν_max_ (cm^-1^): 3132 (Ar C-H), 2862 (Aliphatic C-H), 1602 (C = N); ^1^HNMR (DMSO-*d*_*6*_*,* 400 MHz) δ, ppm: 8.69 (1H, s, ArH); 7.98 (1H, s, ArH); 7.85 (1H, d, J = 8, ArH); 7.79 (2H, d, J = 8, ArH); 7.41–7.44 (2H, m, ArH); 7.19–7.26 (5H, m, ArH); 6.26 (1H, s, CH); 5.39 (1H, d, J = 12, CH_2_); 5.22 (1H, d, J = 16, CH_2_); 2.32 (1H, s, CH_3_). ^13^C-NMR (DMSO-*d*_*6*_, 100 MHz) δ (ppm): 163.52, 161.31, 161.09, 146.25, 146.19, 144.64, 140.95, 138.73, 137.16, 131.46, 129.73, 129.19, 129.11, 126.84, 125.13, 123.72, 120.40, 115.97, 115.75, 110.62, 84.52, 72.80, 21.31. ES^+^ (m/z) = 428.4 (M + H)^+^

*2-(1-(4-Fluorophenyl)-1,3-dihydroisobenzofuran-5-yl)-6-(4-methoxyphenyl)imidazo[2,1-b][1,3,4]thiadiazole* (**3e**): Off white solid. 60% yield. IR: ν_max_ (cm^-1^): 3148 (Ar C-H), 2833 (Aliphatic C-H), 1602 (C = N), 1487 (C = C); ^1^HNMR (DMSO-*d*_*6*_*,* 400 MHz) δ, ppm: 8.63 (1H, s, ArH); 7.98 (1H, s, ArH); 7.81–7.85 (3H, m, ArH); 7.41–7.45 (2H, m, ArH); 7.21–7.26 (3H, m, ArH); 7.00 (2H, d, J = 8, ArH); 6.26 (1H, s, CH); 5.39 (1H, d, J = 12, CH_2_); 5.22 (1H, d, J = 16, CH_2_); 3.79 (1H, s, CH_3_). ^13^C-NMR (100 MHz, CDCl_3_) δ (ppm): 162.99, 160.53, 159.57, 158.31, 145.66, 144.57, 143.85, 139.66, 135.99, 135.96, 129.20, 127.87, 127.79, 125.57, 125.48, 125.38, 122.19, 118.41, 114.77, 114.55, 113.16, 107.38, 84.36, 71.75, 54.31. ES^+^ (m/z) = 444.5 (M + H)^+^.

### Molecular docking

The interaction of the synthesized target compounds (ligands) with the protein and its poses were studied using the Schrodinger suite. Initially, Ligprep was used to prepare ligands followed by protein preparation by importing the crystal structure of E.coli Fabh (PDB ID: 5BNS) and human HDAC7 (PDB ID: 3ZNR) from the protein data bank (PDB). A grid was generated using receptor grid generation tool which is an essential step for ligand docking. ADME properties of the derivatives were also established using the Qikprop tool.

### Antimicrobial activity

The minimum inhibitory concentration (MIC) of the synthesized derivatives was determined using serial dilution method. The microorganisms studied includes *S. aureus* MTCC 3160 (bacterial strain)*, M. smegmatis* MTCC 944 (bacterial strain), *E. coli* MTCC 1687 (bacterial strain) and *C. albicans* MTCC 7523 (fungal strain)*.* For the evaluation, 10^2^ fold dilutions of 0.5 McFarland microbial cultures were prepared; the samples were dissolved in DMSO (1 mg/mL). In a 96 well plate, each well containing different concentration of test compounds, nutrient broth and diluted bacterial or fungal culture were mixed and incubated overnight in room temperature. Control microbial culture was processed in triplicates similar to the samples without any inhibitors and considered this as 100% growth (negative control). The antibacterial standard (ciprofloxacin) and antifungal standard (fluconazole) were also diluted serially in the wells A-H. After 24 h of incubation, to each well 10 μL of resazurin dye (0.001%) was added to find out the MIC. The antimicrobial activity protocol was followed according the protocol reported earlier^39^.

### Anticancer activity

The synthesized derivatives were evaluated for cytotoxicity against MCF-7 cell line (NCCS, Pune) by Sulforhodamine B (SRB) assay. The human breast cancer cells (MCF-7) were cultured in Dulbecco’s Modified Eagle’s Medium (DMEM) (Sigma Aldrich, India) supplemented with 10% fetal bovine serum (FBS) (Invitrogen), 1% penicillin/streptomycin (Sigma Aldrich, India) and incubated in a humidified incubator with 5% CO_2_ at 37 ˚C. SRB assay was performed using the method described by Vichai et al. MCF-7 cells were seeded at a density of 5000 cells per well in a 96-well plate and incubated for 48 h at 37 ˚C in an incubator with a 5% CO_2_ supply. After the monolayer formation of the cells, they were treated with different concentrations (3.125 μM, 6.25 μM, 12.5 μM, 25 μM, 50 μM and 100 μM) of Cisplatin (standard drug) and the test compounds, incubated for 48 h. The cells were then fixed by incubating with ice-cold 10% trichloroacetic acid (TCA) (SRL) for one hour at 4 ˚C. After cell fixation, the cells were washed with water three times to remove extra TCA, followed by air drying of plates. The dried plates were stained with SRB dye (Sigma Aldrich, India) for 30 min in a dark environment and then rinsed with 1% acetic acid (Sigma Aldrich, India) to remove the extra dye. The bound dye was then dissolved in 10 mM tris base (Himedia), and the absorbance was measured at 530 nm^40^.

### Molecular dynamics (MD) simulation

MD simulations were carried out for anticancer protein HDAC7 (PDB: 3ZNR) with ligand **3c**. The protein–ligand complex (HDAC7-3c) was established after ligand docking. System builder module of Desmond was used to build the model where TIP3P was chosen as solvent model and the shape of the box was kept as orthorhombic and the volume was minimized with 10 Å each distance dimension. 0.15 M NaCl salt concentrations were added and the system was auto neutralized by recalculating the required ions and was run with OPLS4 force field. Simulation was performed using molecular dynamics module by loading from workspace for a period of 100 ns. The model system was relaxed before simulation with normal pressure temperature (NPT) at 1.01325 bar pressure and 300 K temperature using Martina-Tobias-Klein method as barostat and Norse-Hoover chain method as thermostat respectively^41^.

## Conclusion

A novel series of five imidazo[2,1-b][1,3,4]thiadiazole derivatives was synthesized, characterized, and biologically evaluated. Spectroscopic analysis, including NMR and mass spectrometry, confirmed the successful formation of the derivatives. Antimicrobial screening revealed comparable activity across all derivatives, regardless of substituents. Notably, anticancer evaluation against the MCF-7 cell line demonstrated promising results, with three derivatives exhibiting significant activity. Among these, derivative **3c**, bearing a nitro substituent, showed the highest potency with an IC_50_ of 35.81 μM, followed by derivatives **3a** and **3d** with IC_50_ values of 52.62 μM and 61.74 μM, respectively. Molecular dynamics simulations of **3c** with protein (PDB: 3ZNR) indicated strong stability, underscoring its potential. These findings suggest that this series holds promise as future drug candidates, on further investigation.

## Electronic Supplementary Material

Below is the link to the electronic supplementary material.

Supplementary Material 1

## Data Availability

Data is provided within the manuscript or supplementary information files.
