# Supplementary material for: In vitro and in silico insights into antimicrobial and anticancer activities of novel imidazo[2,1-b][1,3,4]thiadiazoles
Source: Sci Rep. 2024 Dec 30;14:31994. doi: 10.1038/s41598-024-83498-x (PMC11685468; doi:10.1038/s41598-024-83498-x)
Supplement: Supplementary file 1 — Supplementary Material 1 [file 41598_2024_83498_MOESM1_ESM.docx]

# Supplementary Data

***In Vitro* and *In Silico* Insights into Antimicrobial and Anticancer Activities of Novel Imidazo[2,1-b][1,3,4]thiadiazoles**

# FTIR data of derivatives


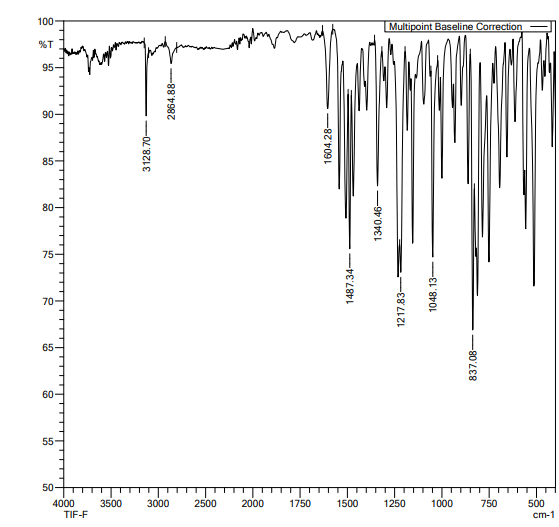


Figure S1: IR spectrum of compound 3b


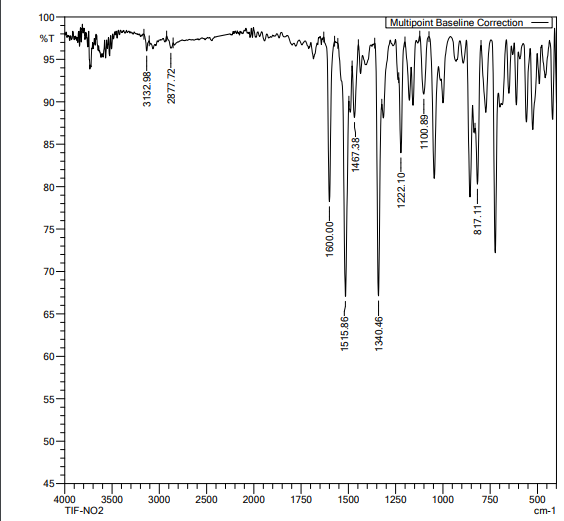


Figure S2: IR spectrum of compound 3c


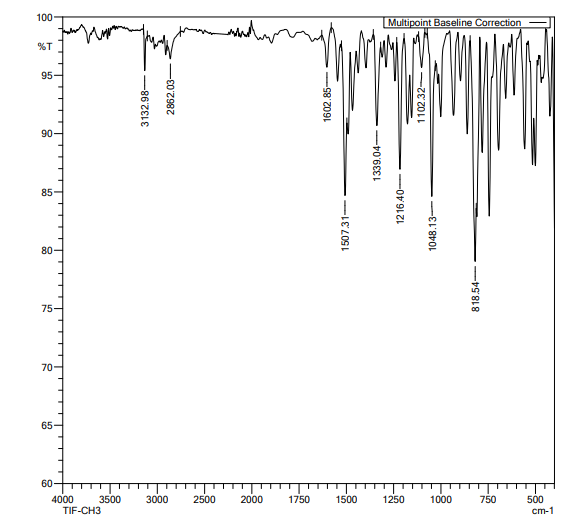


Figure S3: IR spectrum of compound 3d

**
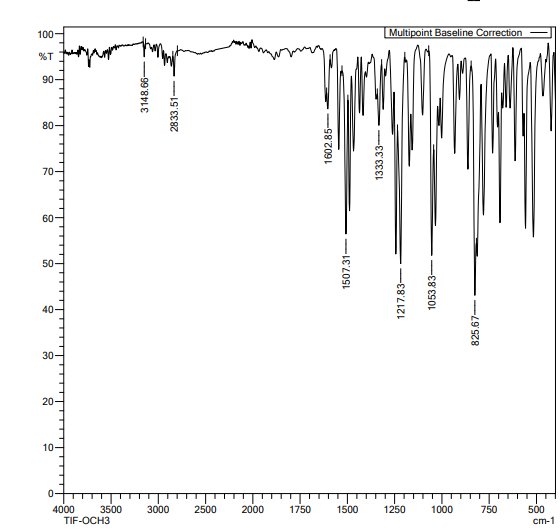
**

Figure S4: IR spectrum of compound 3e

^1^H-NMR data of derivatives


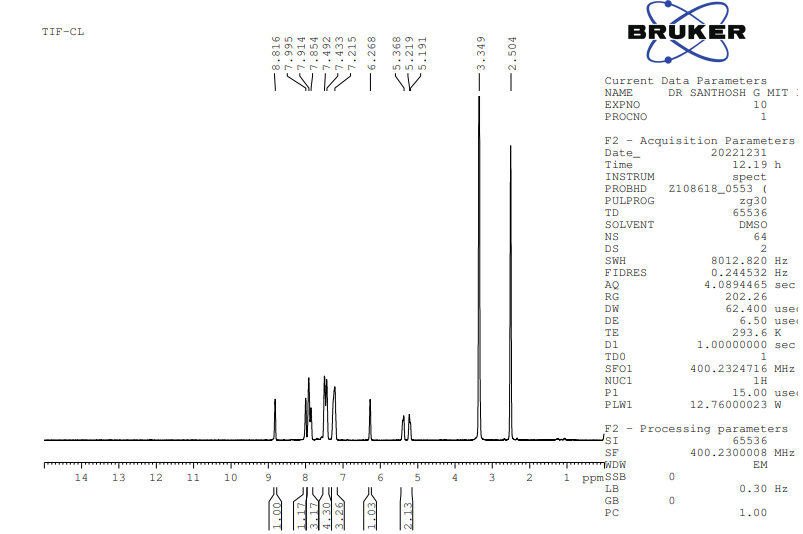


Figure S3:^1^HNMR spectrum of 3a


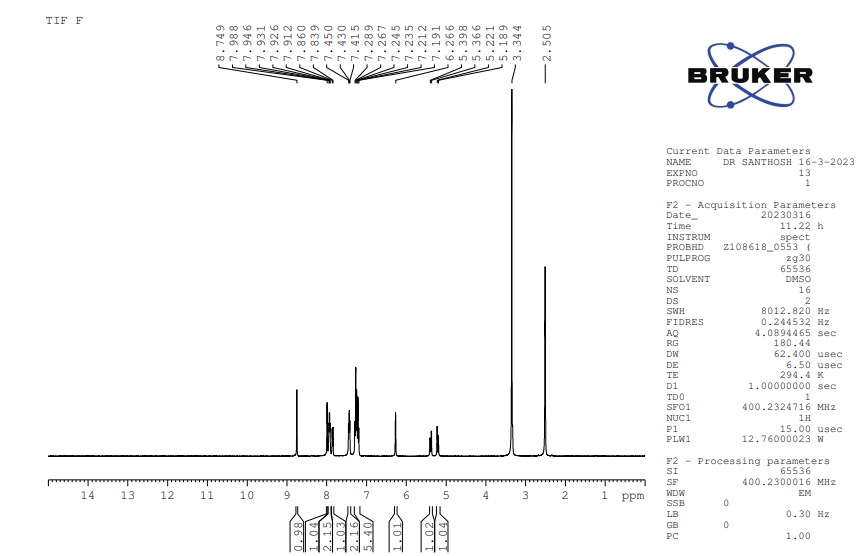


Figure S6: ^1^HNMR spectrum of 3b


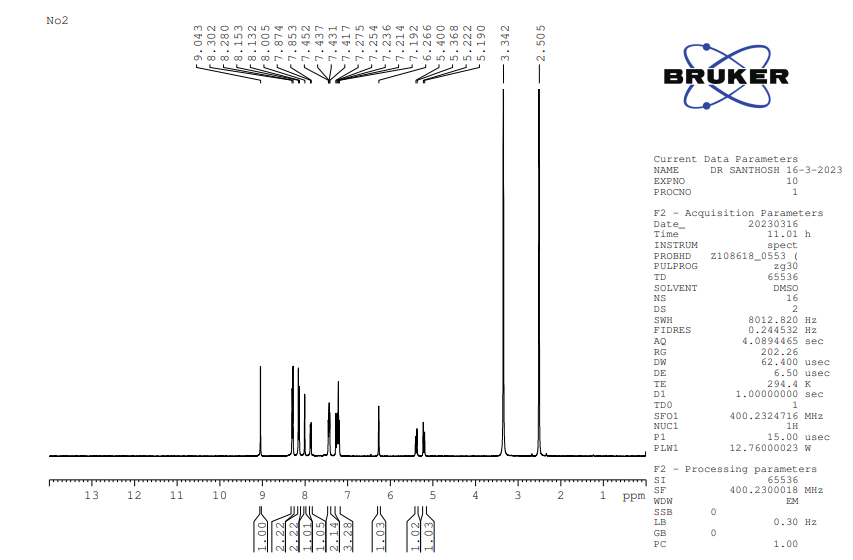


Figure S7: ^1^HNMR spectrum of 3c


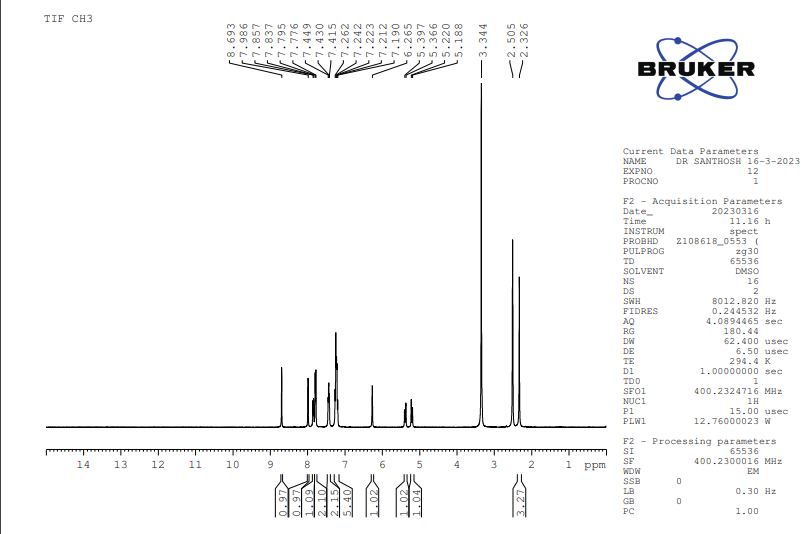


Figure S8: ^1^HNMR spectrum of 3d


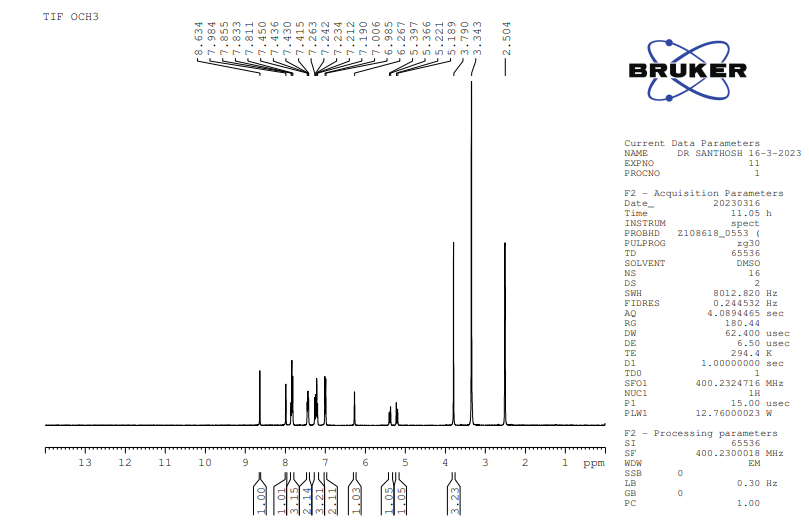


Figure S9: ^1^HNMR spectrum of 3e

# ^13^C-NMR data of derivatives


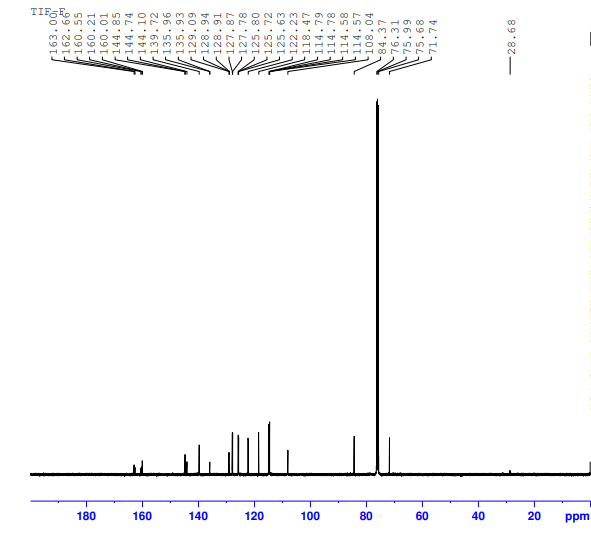


Figure S10: ^13^C NMR spectrum of 3b


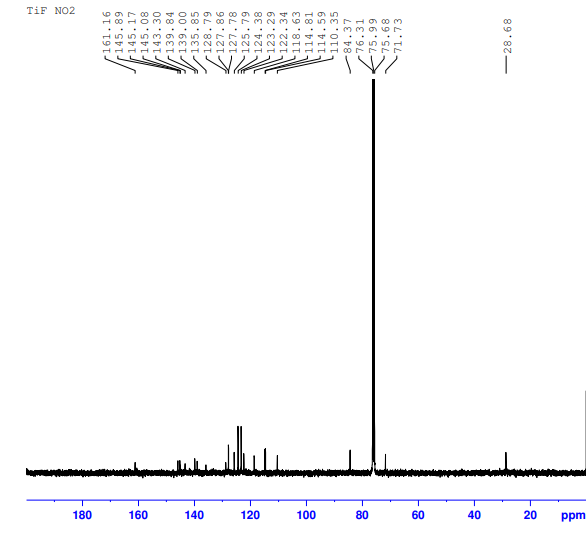


Figure S11: ^13^C NMR spectrum of 3c


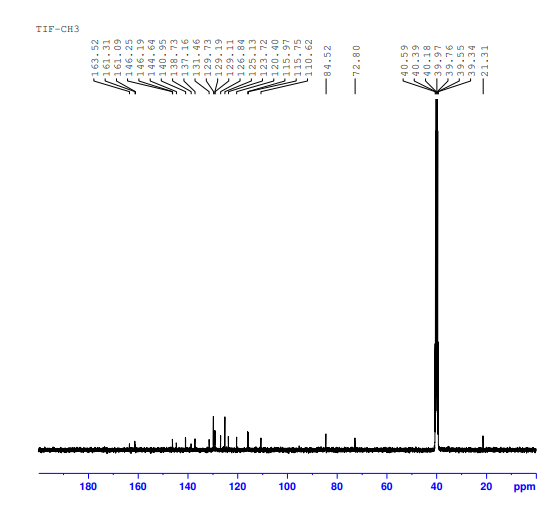


Figure S12: ^13^C NMR spectrum of 3d


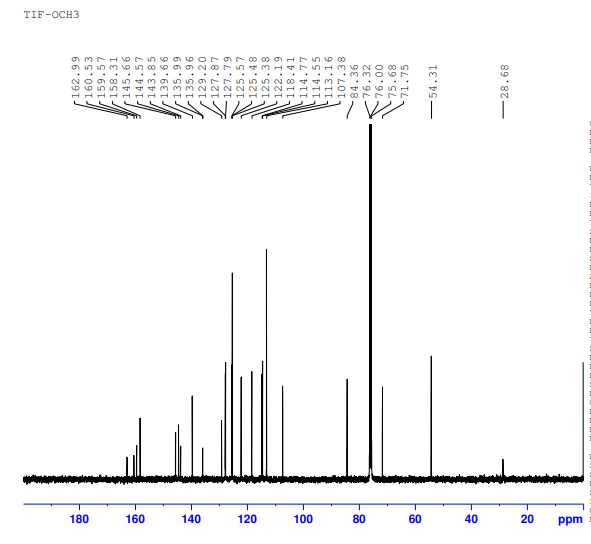


Figure S13: ^13^C NMR spectrum of 3e

# ESI-MS data of the derivatives


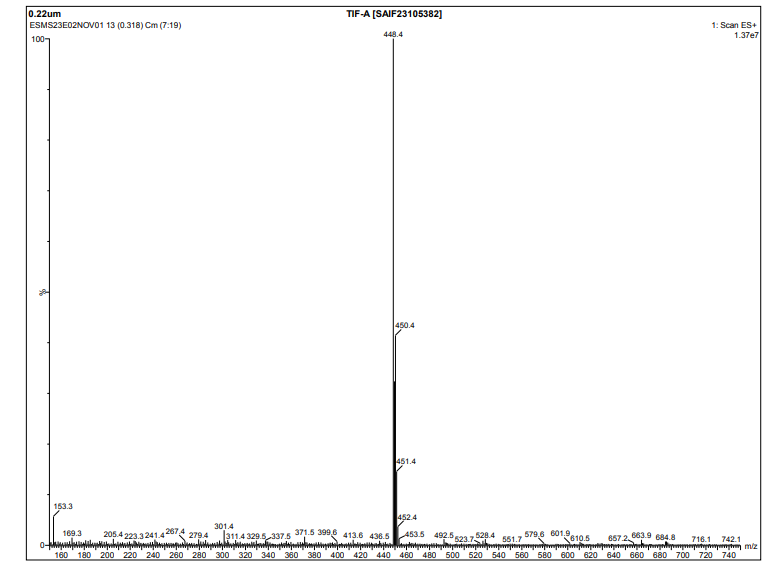


Figure S14: HRMS of 3a


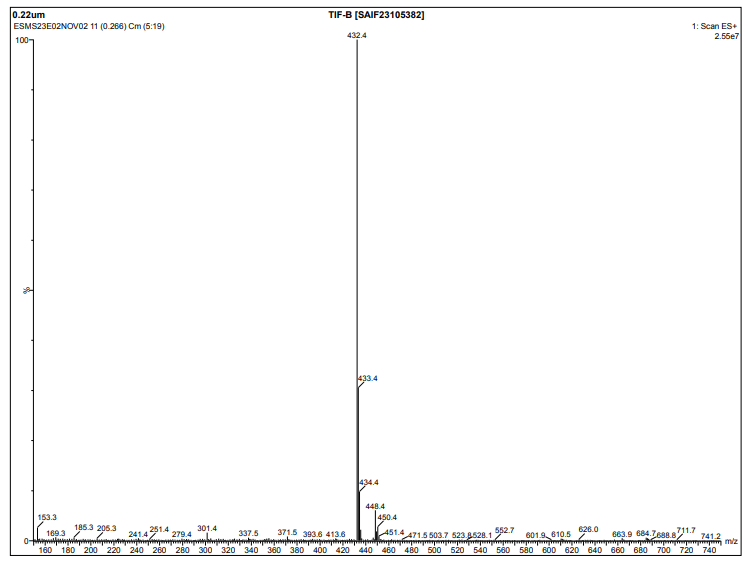


Figure S13: HRMS of 3b

**
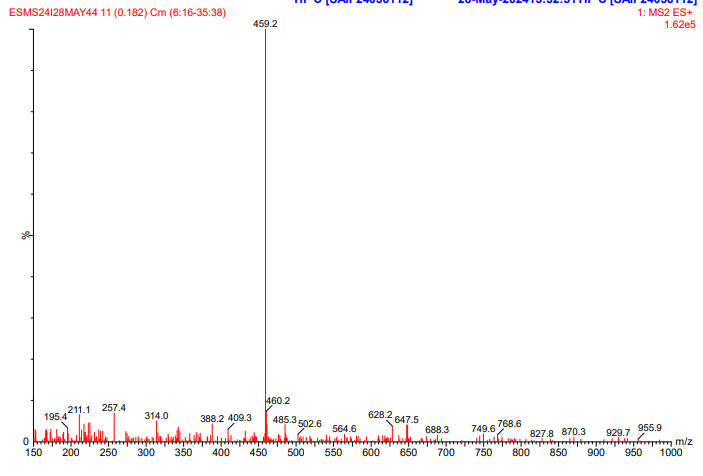
**

Figure S16: HRMS of 3c

**
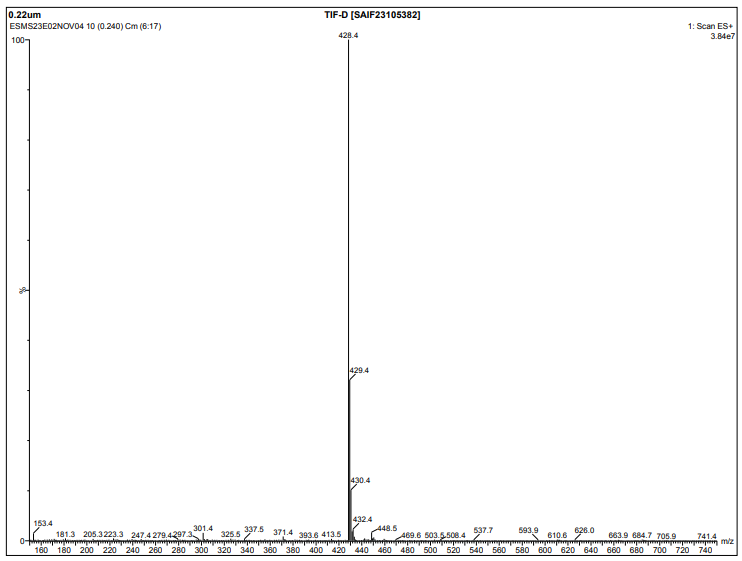
**

Figure S17: HRMS of 3d


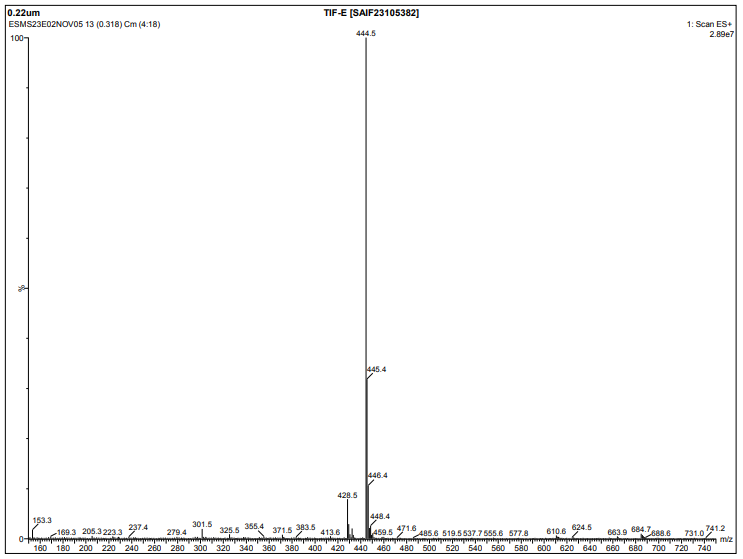


Figure S18: HRMS of 3e
